# Self-esteem in adolescents with Angle Class I, II and III malocclusion in a Peruvian sample

**DOI:** 10.1590/2177-6709.21.2.059-064.oar

**Published:** 2016

**Authors:** Karla Florián-Vargas, Marcos J. Carruitero Honores, Eduardo Bernabé, Carlos Flores-Mir

**Affiliations:** 1 Dentist, Private Practice, Trujillo, Peru.; 2 Assistant professor, Universidad Privada Antenor Orrego, Trujillo, Peru.; 3 Senior lecturer, King's College London Dental Institute, Division of Population and Patient Health, London, United Kingdom.; 4 Associate professor and Head of the Division of Orthodontics, University of Alberta, Edmonton, Canada.

**Keywords:** Self-esteem, Malocclusion classification, Adolescents.

## Abstract

**Objective:**

To compare self-esteem scores in 12 to 16-year-old adolescents with different Angle malocclusion types in a Peruvian sample.

**Material and Methods:**

A cross-sectional study was conducted in a sample of 276 adolescents (159, 52 and 65 with Angle Class I, II and III malocclusions, respectively) from Trujillo, Peru. Participants were asked to complete the Rosenberg Self-Esteem Scale (RSES) and were also clinically examined, so as to have Angle malocclusion classification determined. Analysis of covariance (ANCOVA) was used to compare RSES scores among adolescents with Class I, II and III malocclusions, with participants' demographic factors being controlled.

**Results:**

Mean RSES scores for adolescents with Class I, II and III malocclusions were 20.47 ± 3.96, 21.96 ± 3.27 and 21.26 ± 4.81, respectively. The ANCOVA test showed that adolescents with Class II malocclusion had a significantly higher RSES score than those with Class I malocclusion, but there were no differences between other malocclusion groups. Supplemental analysis suggested that only those with Class II, Division 2 malocclusion might have greater self-esteem when compared to adolescents with Class I malocclusion.

**Conclusion:**

This study shows that, in general, self-esteem did not vary according to adolescents' malocclusion in the sample studied. Surprisingly, only adolescents with Class II malocclusion, particularly Class II, Division 2, reported better self-esteem than those with Class I malocclusion. A more detailed analysis assessing the impact of anterior occlusal features should be conducted.

## RESUMO

**Objetivo:**

comparar os escores relacionados à autoestima de adolescentes com 12 a 16 anos de idade, portadores de diferentes tipos de má oclusão de Angle, em uma amostra de peruanos.

**Métodos:**

um estudo transversal foi conduzido com uma amostra de 276 adolescentes (159, 52 e 65, respectivamente, com má oclusão de Classe I, II e III de Angle) da cidade de Trujillo, Peru. Os participantes foram instruídos a completar a Escala de Autoestima de Rosenberg (*Rosenberg self-esteem scale*, ou RSES), e também foram submetidos a exames clínicos para determinar qual sua má oclusão, segundo a classificação de Angle. A análise de covariância (ANCOVA) foi utilizada para comparar os escores da RSES dos adolescentes com más oclusões de Classes I, II e III, controlando-se os fatores demográficos dos participantes.

**Resultados:**

as médias dos escores da RSES dos adolescentes com Classes I, II e III foram, respectivamente: 20,47 ± 3,96, 21,96 ± 3,27 e 21,26 ± 4,81. O teste ANCOVA revelou que o escore da RSES dos adolescentes com Classe II foi significativamente maior do que o escore dos adolescentes com Classe I, mas não houve diferenças entre os outros grupos de más oclusões. Uma análise adicional sugeriu que podem ser somente os adolescentes Classe II, subdivisão 2, que apresentam autoestima mais elevada, quando comparados aos adolescentes Classe I.

**Conclusão:**

esse estudo mostrou que, no geral, a autoestima dos adolescentes incluídos na amostra estudada não variou em função da má oclusão apresentada. Inesperadamente, apenas os adolescentes com Classe II - especialmente os com Classe II, subdivisão 2 - relataram apresentar uma autoestima mais elevada do que os adolescentes com Classe I. Uma análise mais detalhada deve ser conduzida, avaliando qual característica oclusal da região anterior tem mais impacto.

## INTRODUCTION

The physical, social and psychological consequences of a malocclusion have been topics of research for a long time. However, the related evidence is still conflicting. Although studies generally report an association between malocclusion and quality of life scores, the strength of evidence is relatively low. There is a need for standardized methods to enhance comparability between studies.^1^ ^,^ ^2^ ^,^ ^3^ In addition, other subjective domains, such as well-being, happiness and self-esteem, have remained largely unexplored in relation to malocclusion. 

Self-esteem can be defined as the perception of one's own ability to effectively master or deal with the surrounding environment, and it is affected by the reactions of others towards an individual.^4^ ^,^ ^5^ ^,^ ^6^ It was initially claimed that facial features, especially those related to oral aesthetics, may have a high potential to influence self-esteem, especially during life stages when there is intense social and affective interaction.^7^ However, the scarce literature on the subject provides conflicting evidence, with some authors arguing that malocclusion affects patients' self-esteem;^8^ ^,^ ^9^ while others report weak to nonsignificant effects of malocclusion^10^ or orthodontic treatment.^11^ ^,^ ^12^ ^,^ ^13^ Reasons are probably related to the multifactorial nature of self-esteem and how individuals may weight individual factors differently. Further research is needed to shed some light onto this research area. There have been no studies reported in Peruvian people, in spite of differences that would need additional investigation, specifically to this population.

The aim of this study was to compare self-esteem scores in 12 to 16-year-old adolescents with different types of Angle malocclusions in a Peruvian sample. It was hypothesized that adolescents with Class II and III malocclusions would report lower levels of self-esteem than those with Class I malocclusion.

## MATERIAL AND METHODS

### Study sample

A cross-sectional study was conducted with a sample of 276 adolescents aged between 12 to 16 years old, recruited from a typical public school in the Porvenir District, Trujillo, Peru. The sample was selected from a population of 1083 students (550 males and 533 females), with the aid of a stratified random sampling method proportional to each level of study (332, 220 199, 193 and 139 students, from first to fifth grade, respectively). The study included adolescents with permanent dentition; and excluded those who had craniofacial syndromes or congenital malformations, any missing tooth (except for third molars) and had received or were undergoing orthodontic treatment. 

A minimum sample size of 156 adolescents (52 comprising each one of the three Angle malocclusion groups) was required to estimate a mean difference in the Rosenberg Self-Esteem Score equal to or greater than 0.55 units between two of those groups, with an 80% statistical power, 95% confidence level and a common standard deviation of 1 unit. 

The study protocol was approved by the Stomatology Permanent Research Committee of *Universidad Privada Antenor Orrego* (Trujillo, Peru). A written informed consent form from the participants' parents and an informed assent form from the adolescents were obtained before participation.

### Data collection

Data were collected with the aid of a self-administered questionnaire and clinical examinations. The ten-item Rosenberg Self-Esteem Scale (RSES) was used to assess participants' global self-esteem.^5^ ^,^ ^14^ Responses to the ten items were scored using a four-point scale ranging from 0 (strongly disagree) to 3 (strongly agree) with items 2, 5, 6, 8, and 9 reverse scored. Higher scores indicate higher self-esteem (possible scores range from 0 to 30). The Spanish version of the RSES was used for this study and showed good psychometric properties (validity and reliability) in a similar adolescent population.^15^ Cronbach's alpha of the RSES was 0.70 in this sample. 

Participants' type of malocclusion was classified according to Angle's classification which is mainly based on the anteroposterior position of the mandibular first permanent molar in relation to the maxillary first permanent molar, and complementarily on the anteroposterior position of anterior teeth. During clinical examination, participants were classified as having Class I (both molars are in good anteroposterior relationship), Class II (mandibular molar is posteriorly positioned) or Class III malocclusion (mandibular molar is anteriorly positioned).^16^ One trained examiner carried out all clinical examinations in a separate room within the school facilities, under natural light and using a tongue depressor. Ten individuals were re-examined after a week for reliability. Kappa values for intra- and inter-reliability were 1.00 and 0.85, respectively.

### Statistical analysis

RSES total score showed a negatively skewed distribution, suggesting the use of nonparametric tests. However, we used analysis of covariance (ANCOVA) to compare the total score, as ANCOVA has several advantages over nonparametric tests.^17^ It allows compensating for multiple comparisons by using an omnibus test, controlling for categorical and continuous confounders (adolescents' sex and age in years, respectively) and testing for interactions among explanatory variables.^17^ ^,^ ^18^ Post-hoc comparisons between pairs of malocclusion groups were conducted by Scheffé's test and only if the omnibus test was statistically significant. A logistic regression analysis was used to evaluate the influence of malocclusion on low self-esteem.

## RESULTS

A total of 276 12 to 16-year-old adolescents (141 girls and 135 boys) participated in the present study. Their mean age was 14.2 ± 1.3 years, with a quarter of the sample being 14 years old. According to Angle's classification, 57.6% of the sample had Class I malocclusion, 18.9% had Class II malocclusion and 23.5% Class III malocclusion (Table 1).

**Table 1:** Description of the sample of adolescents (n = 276).

Characteristic		n	%
Sex	Girls	141	51.1
	Boys	135	49.9
Age	12 years	29	10.5
	13 years	58	21.0
	14 years	70	25.4
	15 years	62	22.5
	16 years	57	20.6
Angle's classification	Class I	159	57.6
	Class II	52	18.9
	Class III	65	23.5

Table 2 shows the mean scores for RSES individual items and the total score. The mean RSES total score was 20.93 ± 4.09; range = 0 - 30. There was no floor effect (minimum possible score), but four individuals had the maximum possible score (ceiling effect). Mean scores for RSES individual items ranged between 1.07 for the item "I wish I could have more respect for myself," and 2.53 for the item "I am able to do things as well as other people." 

**Table 2:** Item and total scores for Rosenberg Self-Esteem Scale in the sample (n = 276).

Item	Mean	SD	Range
On the whole, I am satisfied with myself	2.42	0.60	0-3
At times, I think I am no good at all	2.38	0.58	0-3
I feel that I have a number of good qualities	2.25	0.65	0-3
I am able to do things as well as most other people	2.53	0.61	0-3
I feel I do not have much to be proud of	2.47	0.62	0-3
I certainly feel useless at times	1.75	0.90	0-3
I feel that I am a person of worth, at least on an equal plane with others	2.24	0.87	0-3
I wish I could have more respect for myself	1.07	0.95	0-3
All in all, I am inclined to feel that I am a failure	2.05	0.92	0-3
I take a positive attitude toward myself	1.77	1.01	0-3
Self-esteem total score	20.93	4.09	0-30

**Table 3:** Comparison of self-esteem scores by malocclusion type (n = 276).

Malocclusion	n	Mean	SD	(95% CI)
Class I	159	20.47^a^	3.96	(19.85 - 21.09)
Class II	52	21.96^a^	3.27	(21.05 - 22.87)
Class III	65	21.26	4.81	(20.07 - 22.45)
*p* value		0.048		

ANCOVA was used for comparison, controlling for adolescents' sex and age (continuous form).

Superscripts indicate which groups were significantly different (Scheffé's post-hoc test was used).

There were significant differences in the total self-esteem score between the three Angle's malocclusion groups (ANCOVA test, *p* = 0.048). Post-hoc comparisons showed that adolescents with Class II malocclusion had a significantly higher total self-esteem score than those with Class I malocclusion (21.96 *versus* 20.47 units). There were no significant differences between other malocclusion groups. Finally, the two-way interaction terms between malocclusion group and sex, and malocclusion group and age, were not significant (*p* > 0.05 in both cases).

In further exploratory analysis, due to limited numbers of participants with Class II, Divisions 1 and 2 malocclusion (30 and 22 individuals, respectively), there was significant difference in the RSES score between Class II, Division 2 (Mean = 22.59, SD = 2.50) and Class I malocclusion groups (independent group t-test, *p* = 0.001), but not between Class II, Division 1 (Mean = 21.50, SD = 3.70) and Class I malocclusion groups (independent group t-test, *p* = 0.172). 

A logistic regression analysis was used to evaluate the influence of malocclusion on low self-esteem. According to this analysis, there was no influence of any type of malocclusion on low self-esteem (R^2^ = 0.01, *p* > 0.05).

## DISCUSSION

Our findings showed that self-esteem scores, as measured by RSES, vary by certain Angle's malocclusion groups. Contrary to our hypothesis, only adolescents with Class II malocclusion reported higher self-esteem when compared to those with Class I malocclusion. No other groups were found to differ in terms of self-esteem scores. Our findings disagree with previous studies in which adolescents with Class II malocclusion had poorer self-esteem, which was measured by the child self-esteem scale, than those with Class I and III malocclusions.^9^ In further supplemental, but exploratory analysis, we also found that it may be those with Class II, Division 2 malocclusion who have greater self-esteem compared to adolescents with Class I malocclusion.

We could speculate on possible explanations for the present findings. First, Class II, Division 2 malocclusion individuals tend to have a very prominent chin, straight or concave facial profile and strong facial muscular definition.^19^ These features could very well be associated with a more aesthetic facial definition. The problem with this explanation is that prominent chins and strong facial musculature are characteristics that are associated with male facial features and not female ones. 

A second explanation relates to how adolescents' malocclusion was assessed in this study. Angle's classification remains the most commonly used classification of malocclusions and its universal acceptance by the dental profession is evidence of its practicability.^20^ We chose this classification for the relationship it has with the facial profile of the patient,^21^ which impacts on oneself and lay person's perception.^22^ However, Angle's classification system was mainly based on the anteroposterior position of first molars,^16^ and not all anterior dentoalveolar features that are likely to impact lay person's aesthetic preferences. Puzzling in Class II, Division 2, the usual dentoalveolar characteristics are proclined upper laterals, retroclined centrals, deep bite and excessive upper incisor display at smiling. These are not features that are normally aesthetically pleasant.^23^ ^,^ ^24^ Evaluation of more specific features, such as overjet, overbite, dentoalveolar discrepancy, gingival exposure, labial competence and position, among others, may allow for a more precise discrimination and identification of which particular occlusal traits are linked to poor and high self-esteem, respectively. There is some preliminary evidence that crowding of anterior teeth may affect adolescents' self-esteem, particularly among girls.^8^

A final explanation is that malocclusion considered as an anteroposterior classification has no actual impact on adolescents' self-esteem. This is reflected by the fact that the differences found between Class II and Class I malocclusions may be statistically significant, but not clinically important. RSES ranges from 0 to 30 units, with values between 15 and 25 considered normal. Values below 15 and above 25 are indicative of low and high self-esteem, respectively.^5^ ^,^ ^14^ ^,^ ^25^ All malocclusion groups had, on average, values within the normal range, suggesting that differences between groups may not be of clinical importance. Prior research has shown that malocclusion and orthodontic treatment have no impact on self-esteem.^10^ ^-^ ^13^ Nevertheless, low self-esteem has been associated with the aesthetic impact of malocclusion, and that it significantly affects the quality of life of schoolchildren.^26^ In addition, an anteroposterior classification of malocclusion does not consider the severity of malocclusion. This aspect could have affected the results, clinical significance and implications on the priority need for treatment, specifically when considering public health policies and resource allocation. 

Some limitations of this study need to be discussed. First, all adolescents included in this study had some type of malocclusion, and, therefore, had some degree of need for orthodontic treatment. Having an alternative comparison group with no malocclusion might have produced different results. The absence of a control group could provide possible implications on the outcomes found, mainly the possibility to compare the results with a normal occlusion. Furthermore, self-esteem could be influenced by several factors; a person may have high self-esteem in his or hers working life and low self-esteem in his or hers personal life. Also self-esteem could have hereditary characteristics, and genetics could play a role on it too. It is difficult to identify the pure contribution of malocclusion on the self-esteem of individuals. It would be necessary to consider these aspects in further investigations.

Second, we used RSES to measure adolescents' self-esteem and it is possible that results may be different if a different scale was used. However, RSES is one of the most widely used measures of global self-esteem in social sciences.^25^ The popularity of RSES is due in part to its good psychometric properties, simplicity and brevity (only 10 questions that can be completed within 1-3 minutes). More importantly, it has been adapted to several languages, including Spanish,^16^ and has been used in different populations and settings.^25^ ^,^ ^27^ Overall, further research should evaluate self-esteem by means of multiple instruments and considering anterior teeth features, with and without malocclusion also classified in transversal and vertical ways.

## CONCLUSION

This study shows that self-esteem may vary according to adolescents' malocclusion in the sample studied. Adolescents with Class II malocclusion (and more specifically, Class II, Division 2) report better self-esteem than those with Class I malocclusion. No differences were found between other malocclusion groups.
